# Antimicrobial Effects of Cannabidiol (CBD)-infused Lozenges against *Streptococcus mutans* in Oral Health

**DOI:** 10.1590/0103-644020245988

**Published:** 2024-12-06

**Authors:** Jila Torabi, Henrique Pedro Soares Luis, Gohar Mkrtchyan, Shohreh Derakhshan Alavijeh, Sameen Dezfoli, Michelle Hurlbutt

**Affiliations:** 1 University of Lisbon, Faculty of Dental Medicine, Lisbon, Portugal; 2 West Coast University Dental Hygiene Program, Anaheim, CA, USA.; 3 Center for Innovative Care and Health Technology (ciTechcare), Polytechnic of Leiria, Leiria, Portugal.

**Keywords:** CBD, cannabidiol, streptococcus mutans, oral biofilm, dental caries

## Abstract

Cannabidiol presents several benefits, including but not limited to its analgesic, antioxidant, anti-inflammatory, antimicrobial, anti-pruritic, and anti-cancer properties. In this clinical trial, the antimicrobial impact of CBD-infused lozenges on *Streptococcus mutans* was examined using quantitative polymerized chain reaction (qPCR) bacterial analysis. This clinical trial involved 30 dental hygiene and nursing students who met the inclusion criteria participated in the study and were divided into two groups: experimental and control. The experimental group was given CBD-infused lozenges for 15 days, while the control group received sugar-free candy. Participants consumed one CBD-infused lozenge (300 mg) daily for 15 days, allowing it to dissolve slowly in the mouth for gradual absorption. The study focused on measuring changes in the salivary levels of *Streptococcus mutans* using quantitative polymerized chain reaction (qPCR) tests. Saliva samples were collected, and DNA extracted for qPCR analysis, assessing the bacterial load. The results, analyzed using a t-test, showed a significant decrease in *Streptococcus mutans* levels in the experimental group compared to the control group, with a statistically significant difference (p=0.0299).These findings suggest that cannabidiol may effectively reduce *Streptococcus mutans* in saliva, thus potentially helping to lower the risk of tooth decay as a multifactorial disease. This study underscores the potential of cannabidiol in enhancing oral health and calls for further research to explore its therapeutic applications in dental care.

## Resumo

O canabidiol apresenta diversos benefícios, incluindo, mas não se limitando às suas propriedades analgésicas, antioxidantes, anti-inflamatórias, antimicrobianas, antipruriginosas e anticancerígenas. Neste ensaio clínico, o impacto antimicrobiano de pastilhas com infusão de CBD sobre *Streptococcus mutans* foi examinado por meio de análises bacterianas utilizando reação em cadeia da polimerase quantitativa (qPCR). Este ensaio clínico envolveu 30 estudantes de higiene dental e enfermagem que atenderam aos critérios de inclusão e foram divididos em dois grupos: experimental e controle. O grupo experimental recebeu pastilhas com infusão de CBD por 15 dias, enquanto o grupo controle recebeu balas sem açúcar. Os participantes consumiram uma pastilha com infusão de CBD (300 mg) diariamente durante 15 dias, permitindo que ela se dissolva lentamente na boca para absorção gradual. O estudo focou em medir as mudanças nos níveis salivares de *Streptococcus mutans* utilizando testes de qPCR. Amostras de saliva foram coletadas, e o DNA foi extraído para análise de qPCR, avaliando a carga bacteriana. Os resultados, analisados por meio de teste t, mostraram uma diminuição significativa nos níveis de *Streptococcus mutans* no grupo experimental em comparação ao grupo controle, com uma diferença estatisticamente significativa (p=0,0299). Esses achados sugerem que o canabidiol pode reduzir efetivamente o *Streptococcus mutans* na saliva, ajudando potencialmente a diminuir o risco de cárie dentária como uma doença multifatorial. Este estudo destaca o potencial do canabidiol na melhora da saúde bucal e sugere a necessidade de mais pesquisas para explorar suas aplicações terapêuticas nos cuidados odontológicos.

## Introduction

In recent years, consumer interest in herbal and traditional medicine has increased due to its perceived effectiveness, natural appeal, and lower costs. The World Health Organization (WHO) has recognized this trend and supports integrating traditional medicine into healthcare through its 2014-2023 strategy ^1^.

Cannabidiol (CBD), a cannabinoid from *Cannabis sativa*, is gaining recognition for its potential in treating various illnesses ^2^. *Cannabis sativa L*., with its subspecies, *sativa ssp.* and *indica ssp.*, has long been used in medicine illnesses like cancer, the nervous system, and inflammatory ailments ^3^, with marijuana and hemp differing in THC and CBD levels ^4^. A 2020 report by McGregor et al. noted that CBD products are widely available online without prescription in several countries, including the US, UK, and Japan ^5^. CBD has a broad therapeutic spectrum, including analgesic, antioxidant, anti-inflammatory, and antimicrobial properties ^2^. Research indicates that cannabidiol (CBD) exhibits antibacterial properties against *Streptococcus mutans (S.mutans)* . In a study conducted by Avraham et al. (2023), combining CBD with triclosan, antibacterial activity and suppression of dental biofilm formation was demonstrated ^6^. Similarly, Barak et al. (2022) found that CBD at a concentration of 5 µg/mL reduced *S. mutans* biofilm biomass, suggesting its potential as an anti-caries agent ^7^.

Dental caries and periodontal diseases are primarily caused by dental plaque, but they are multifactorial, influenced by factors like oral microflora. Managing caries requires a comprehensive approach, addressing biological, behavioral, psychological, and environmental determinants ^8^. Oral health practitioners develop preventive strategies tailored to individual risk profiles ^9^. *Streptococcus mutans*, a key bacteria in caries development, adheres to teeth through a sucrose-dependent mechanism, facilitating colonization ^10^. This process facilitates the adhesion and colonization of microbial entities on the dental surface.

Periodontal disease, including gingivitis and periodontitis, is an inflammatory condition affecting the tissues around the teeth ^11^, and is the 11th most prevalent condition globally, contributing to tooth loss. Like dental caries, it is biofilm-driven and influenced by various systemic, local, and environmental factors. The disease develops when subgingival biofilm shifts from symbiotic to dysbiotic, triggering an inflammatory response that damages periodontal tissues ^12^. Plaque-induced gingivitis represents a reversible form of periodontal disease, triggered by dental biofilms. The elimination of such biofilms can revert the tissues to a healthy state.

CBD’s anti-inflammatory properties make it a viable candidate for managing inflammation-related oral diseases, oral and salivary gland cancers, and burning mouth syndrome, while also reducing dental anxiety and supporting oral hygiene ^2^. Özdemir et al. (2014) and Morissette's studies highlighted CBD's role in reducing inflammation in periodontal diseases ^13^. Vasudevan et al. (2020) demonstrated CBD's antibacterial efficacy in mouthwashes and air polishing powders, comparable to chlorhexidine gluconate ^14^ ^,^ ^15^. Studies also indicate CBD's role in successful dental implant osseointegration ^16^ and pain reduction in mouth ulcers ^17^.

Despite growing interest in CBD for oral health, further research is needed to fully understand its effectiveness against oral bacteria and overall oral health impact ^18^. Despite its growing use, there remains a significant gap in understanding CBD's specific antimicrobial effects in the oral cavity, especially against common oral pathogens like *Streptococcus mutans*. This study aims to bridge this gap by focusing on the antimicrobial efficacy of CBD-infused lozenges against *Streptococcus mutans.*

## Materials and Methods

This study was conducted following an approved application from West Coast University's Institutional Review Board (IRB), ensuring adherence to ethical standards for research involving human subjects. Approval was granted (IRB #01202023_torabiDH_CBDloz) on January 20, 2023. This study is registered with the ClinicalTrials.gov Identifier: NCT06301113

A convenience sample of thirty students from West Coast University in Southern California, enrolled in dental hygiene or nursing programs, voluntarily participated. To assess eligibility, a nine-item questionnaire was distributed to potential recruits. This instrument was designed to gather information on the recruits' age, known allergies to ingredients found in candies, dental hygiene practices including recent tooth brushing, dental visits, and the current use of any prescribed oral rinses or antibiotics. The participants, meeting specific inclusion criteria, provided informed consent. Group assignment was determined through a simple random sampling method. The randomization process for the study participants was done using a simple method where 30 index cards were placed in a bag, 15 marked as "A" and 15 marked as "B." Participants drew a card to determine their group assignment, either Group A or Group B. This ensured that the assignment to either the control or experimental group was random, maintaining the integrity of the study design​. Each participant was given a unique identification number (A1-A15 or B1-B15) to maintain anonymity. The simple card-drawing method ensured that neither the participants nor the researchers knew the group assignment in advance, maintaining the integrity of the randomization process. A post-experiment survey was also conducted to gather demographic data and evaluate participants' regimen adherence.

The study's inclusion criteria specified adults aged 18 years or older who were not under any antibiotic treatment, not using prescribed dental care products like toothpaste or mouthwash, and free from toothache or mouth sores. Eligible participants had not visited a dentist in the preceding 48 hours, consented to participate, were available for scheduled dates, and committed to a 15-day regimen involving the nightly consumption of a candy, allowing it to dissolve naturally in the mouth. Additionally, candidates were required to abstain from alcohol for at least 12 hours prior to saliva collection on the first and fifteenth days and avoid food or drink (except water) for 2 hours before each saliva collection, these guidelines were applied consistently across both collection days to ensure uniformity in the study conditions. The exclusion criteria targeted individuals on or recently off antibiotics within two weeks prior to the study, those with allergies to specific ingredients like broad-spectrum nano hemp extract, isomalt, organic stevia, cherry flavoring, natural coloring, maltitol syrup, or citric acid, as well as those using professional dental care products. Also excluded were individuals with recent dental issues or visits, and those unable to comply with the saliva collection protocol ^19^.

This single-blinded study focused on edible, THC-free CBD products from reputable sources. Selected products were free from sugar alcohol and did not include gel or gummy forms. The study employed a single-blind design, ensuring that the personnel performing the laboratory qPCR analysis were blinded to the treatment group assignments. This was done to prevent any bias in the interpretation of the data. The qPCR analysis was conducted by a single experienced individual who was unaware of which group (control or experimental) the samples belonged to. This blinding was maintained throughout the data collection and analysis phases to ensure the objectivity and integrity of the results. The experimental product was a Cherry-flavored CBD Hard Candy-300 Nano mg CBD, consisting of broad-spectrum nano hemp extract, isomalt, organic stevia, natural flavoring, and coloring (CBD LIVING, Calming Cherry CBD Hard Candy, manufactured in San Diego California, United States). This nano formulation of CBD enhances absorption and bioavailability ^20^. The control product was a sugar-free cherry-flavored candy (Jolly Rancher sugar-free Raspberry-flavored candy, product of Hershey Company, produced in Canada, packed by Secret Candy Shop Store) with maltitol syrup, citric acid, artificial cherry flavor, and FD&C Red #40.

Each participant in Group A received 15 CBD-infused candies, while Group B received 15 sugar-free candies. In the study, participants' adherence was closely monitored through the use of a candy consumption compliance chart, which tracked daily consumption. Participants were instructed to consume one candy each evening and received email reminders on the first and last days of the 15-day regimen to encourage adherence. Participants were instructed to maintain their usual oral hygiene practices, consume one candy each evening for 15 days, and let the lozenge dissolve slowly in their mouth. A survey was conducted post-experiment to assess their experience with the candy, namely candy flavor, aftertaste, stimulation of saliva flow, and ease of use of candy.

The saliva samples of participants in each group were collected pre- and post-consumption of the assigned product on days 1 and 15 following the collection of saliva sample protocols ^19^. Investigators filled fifty-milliliter plastic centrifuge screw-cap vials with 5mL 1xPBS sterile, 0.2 μm filtered phosphate-buffered saline sterile solution to preserve integrity of the specimens. The Solution composition was reported (1X):137 mM sodium chloride (NaCl), 2.7 mM potassium chloride (KCl), 12 mM monosodium phosphate. Vials were labeled appropriately for each participant and time point. Researchers witnessed participants expectorate in their assigned vials and secure the cap during the pre-experiment saliva collection following the collection of saliva samples protocol ^19^.

To collect saliva, participants removed the cap of their pre-labeled vial, let saliva flow into it passively, then securely replaced the cap. They placed the vial, along with a candy consumption chart and collection instructions, in a provided bag. This process was observed by researchers during the initial collection to ensure adherence to protocol. Saliva samples were systematically collected over a seven-day period leading to the trial start, ensuring all participants began concurrently. Detailed guidelines were provided to the participants ^19^, instructing them to collect their saliva samples independently on a designated day. Each participant was furnished with a pre-labeled centrifuge vial. Saliva specimens were subsequently stored at a controlled temperature of 37 degrees Fahrenheit. To ensure uniformity in the experimental conditions, all participants were directed to commence the trial concurrently on a specified start date.

Our research aimed to quantify the relative presence of the *Streptococcus mutans* gene against the backdrop of the overall bacterial population in saliva samples, both pre- and post-intervention. The primer sequences Sm1 (5'-GGT CAG CAA AGT CTG TAA AAG GCT T-3') and Sm2 (5'-GCG GTA GCT CCG GCA CTA AGC C-3') ^21^ were employed to specifically target and amplify a conserved segment of the *Streptococcus mutans* 16s ribosomal RNA gene. Saliva samples were subjected to DNA extraction using the Tri reagent method (SIGMA) ^22^, followed by quantification of the extracted total DNA. Subsequently, 2 ng of DNA extracted from the saliva samples was used as a template for each qPCR reaction. This was applied to quantify the abundance of *Streptococcus mutans* in the saliva, using specific primer sequences to amplify the *S. mutans* 16S ribosomal RNA gene. The quantification of *S. mutans* per milliliter of saliva was achieved by comparing the bacterial gene expression to the total bacterial load, providing a measure of the relative abundance of *S. mutans* in the samples. This methodology was executed on five paired saliva samples, collected from participants at the beginning and conclusion of the study period, which met the quality criteria necessary for qPCR analysis. Each sample was subjected to 40 cycles of amplification using the qPCR technique, with three technical replicates performed for accuracy.

The study measured both primary and secondary outcomes in line with CONSORT guidelines. The primary outcome was the quantification of *Streptococcus mutans* gene expression using qPCR analysis. This was assessed pre- and post-intervention in both the experimental and control groups, where the reduction in *S. mutans* gene expression was the main focus. The secondary outcomes included participants' feedback on the product, such as flavor, side effects, changes in saliva production, and any sensations of calmness or drowsiness. These subjective measures provided insights into participants' experience with the CBD-infused lozenges​.

The statistical analysis was conducted using descriptive statistics to characterize the sample. A two-tailed unpaired t-test was employed to compare the reduction in *S. mutans* gene expression between the experimental group (CBD-infused lozenges) and the control group (sugar-free candy). This allowed for the detection of statistically significant differences post-experiment. Missing data, primarily due to non-compliance with saliva collection, led to the exclusion of eight participants, which could have impacted the study's power.

## Results

**Figure 1:**
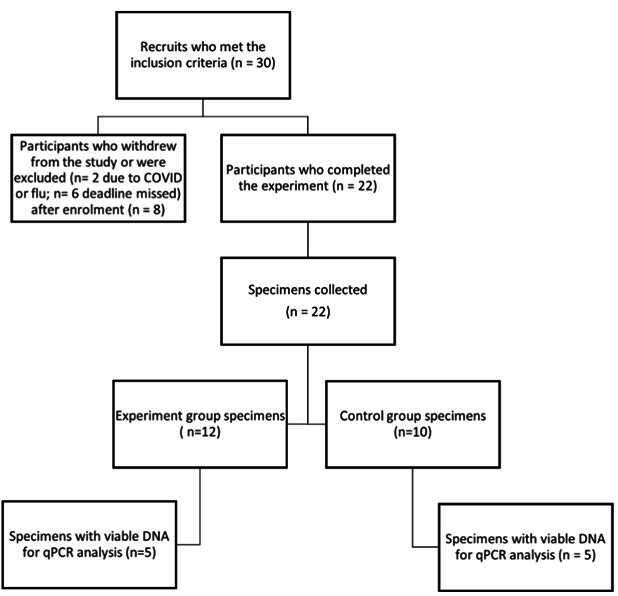
Schematic diagram of the participants' process.

Out of the 30 participants who initially took part in this clinical trial, only 22 were able to complete the study. The remaining eight participants were eliminated from the study due to noncompliance with collecting saliva samples after the experiment and due to illnesses such as COVID or the flu during the study (Figure 1). These factors necessitated their exclusion to ensure the integrity and reliability of the study's results.

The samples were shipped to Azimi labs at Georgia State University, utilizing Biological Substance Category B transfer guidelines for infection control protocol for quantitative Polymerase Chain Reaction (qPCR) analysis.

The demographics of the participants was predominantly female (73.9%) and Asian (39.1%), with ages primarily ranging between 18-24 years (30.4%) and 25-34 years (65.2%). Out of the total participants, 22 fulfilled the inclusion criteria and were divided into two groups: Group A (experimental group) received CBD-infused lozenges, while Group B (control group) was given sugar-free candy. Group A comprised 55% (n=12) of the participants receiving CBD lozenges, and Group B had 45% (n=10) receiving sugar-free candy. Some participants were excluded due to non-compliance with the saliva collection or product receipt. Compliance with saliva collection and regimen adherence was similar in both groups.

Data normality was confirmed using the Shapiro-Wilk test. Participants in Group B, who consumed sugar-free candy, significantly preferred its flavor over that of Group A (p<0.001), with 90.9% (n=10) of Group B individuals expressing a liking for the sugar-free candy taste, compared to 8.3% (n=1) of Group A participants, who consumed CBD-infused lozenges. Analysis from the survey revealed that 58% of participants in Group A noted an increase in saliva production, whereas 100% of individuals in Group B observed a heightened saliva output. Survey results showed that 25% of Group A participants, who ingested the CBD-infused lozenge, reported feelings of drowsiness, whereas no participants in Group B experienced such sensations.

It was noted that the detection of *S. mutans* typically occurred between the 20th and 25th cycles. Detections beyond the 30th cycle were considered to represent a very low copy number of the target gene, thus deemed insignificant for our study.

An unpaired two-tailed t-test was conducted on 5 viable (defined as deemed of sufficient quality for inclusion in the analysis) samples from group A and 5 viable samples from group B, with sufficient 16S rRNA, to compare the change in *Streptococcus mutans* abundance between the two groups post-candy consumption with a 0.05 significance level. Viability of samples was low due to the rigorous standards required for qPCR analysis-standards which are essential for the accuracy and reliability of results.

**Figure 2:**
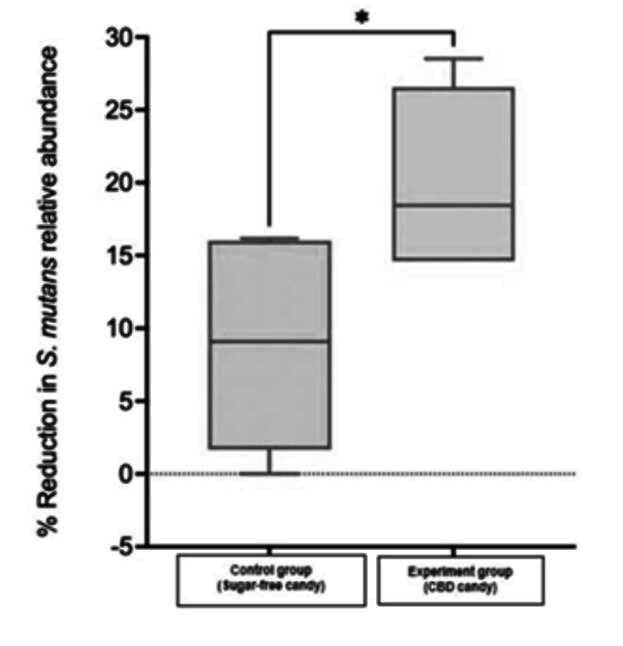
Changes in Streptococcus mutans relative abundance in the experiment versus the control group.*P=0.0299

The analysis revealed a significant reduction in *Streptococcus mutans* abundance in the post-candy consumption group compared to the control group (p=0.0299), indicating the potential antimicrobial effect of the CBD-infused lozenges on oral bacteria (Figure 2). The mean value of the control group was 8.89 and for the experiment group was 20.16 with a 95% confidence interval of (1.41 - 21.12).

**Figure 3:**
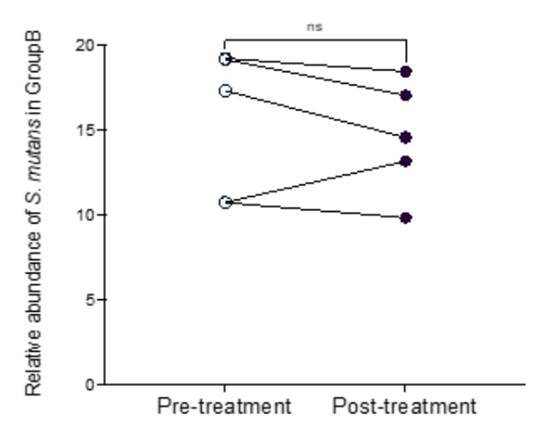
Changes in the relative abundance of S. mutans in the control group (B) ns - not significant.

**Figure 4:**
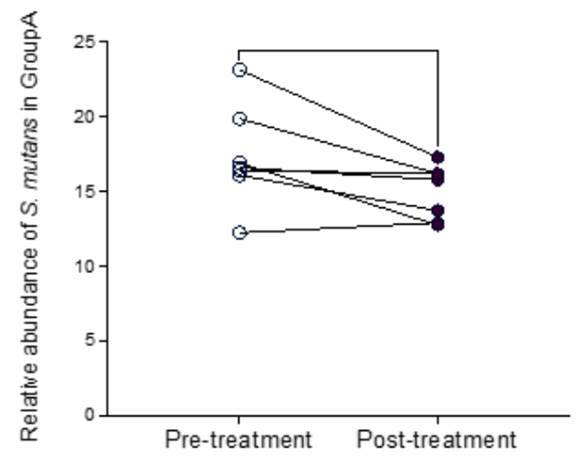
Changes in the relative abundance of S. mutans in the experiment group (A); P>0.05

Figures 3 and 4 illustrate the changes in S. mutans relative abundance before and after treatment in the control and experimental groups, respectively. For group A there was a statistically significant reduction (p<0.05).

## Discussion

The increasing interest in cannabidiol (CBD) for oral health applications reflects the public's increasing preference for natural and herbal health solutions. The anxiolytic, analgesic, and sleep-promoting properties of cannabinoids have been extensively documented. With the CBD-infused lozenge designed to leverage these calming effects, assessing its efficacy in inducing relaxation and sleepiness was crucial. Notably, CBD has been recognized for its extensive therapeutic attributes, including analgesic, antioxidant, anti-inflammatory, and antimicrobial properties, among others ^2^. It is also present in Cannabis, commonly known as marijuana, one of the most frequently used illicit drugs, but it is not impairing. CBD's antimicrobial properties against *Streptococcus mutans*, a bacterium known for its role in dental caries, justify its inclusion in oral health products. As oral health professionals, it is important to understand cannabis' impact on oral health and incorporate questions about cannabis use into patient history, as cannabis users may have an increased risk for oral infections due to its potential immunosuppressive effects ^23^.

This clinical investigation revealed significant differences in the prevalence of target cells, particularly *Streptococcus mutans*, in saliva samples before and after the intake of CBD-infused lozenges compared to sugar-free candy in control groups. Post-consumption results from the CBD group indicated a marked reduction in these target cells. While these findings are promising, it is essential to consider the variable of participant adherence to the usage guidelines, which could affect the bacterial population of *Streptococcus mutans*. The observation that only one participant in the CBD group liked the candy's flavor, combined with just 58% noting increased saliva production, highlights the critical role of flavor in oral healthcare products for ensuring user compliance. This finding underscores the importance of palatability in oral healthcare products, as it can directly affect user adherence. Adequate saliva production is essential for facilitating oral healing, maintaining optimal pH levels in the oral cavity, and preventing oral diseases, including periodontal disease and dental caries. It also emphasizes the necessity for manufacturers to prioritize formulations that enhance saliva production, crucial for maintaining oral health, balancing pH levels, and preventing conditions such as periodontal disease and tooth decay. Additionally, the need for further research to understand CBD's long-term effects is underscored by these results ^24^.

In a study conducted by Gu *et al*. in 2019, the researchers investigated the effects of CBD, cannabidiol (CBN), and THC on *Porphyromonas gingivalis*, *Filifactor alocis*, and *Treponema denticola*. The study found that each of these compounds, CBD, CBN, and THC, reduced the production and release of proinflammatory cytokines, including IL-12 p40, IL-6, and IL-8, which are associated with *Porphyromonas gingivalis*, *Filifactor alocis*, and *Treponema denticola*. Additionally, these compounds were observed to enhance the levels of the anti-inflammatory cytokine IL-10. Notably, CBD specifically disrupted the production of anti-inflammatory cytokines and impacted the CB2 receptor, reducing pain and inflammation ^25^. In 2023 a study demonstrated the beneficial role of a combined triclosan/CBD treatment for protection against dental caries, since the biofilm formation was inhibited, and the concentrations required for the inhibition of *S. mutans* were non-toxic ^6^. An in-vitro study investigating CBD's minimum inhibitory concentrations (MIC) against *Streptococcus mutans* and its minimum biofilm inhibitory concentration (MBIC) against biofilm formation revealed significant findings. Barak et al. 2022 reported that CBD at a concentration of 5μg/ml inhibited *Streptococcus mutans* growth and biofilm development, thus preventing bacteria-induced pH decreases ^7^.

The study's limitations encompass multiple factors, notably the small sample size, lack of diverse demographics, the researchers' challenges in consistently collecting post-experiment specimens on the same day from all participants, and the potential for non-compliance in collecting saliva at home and adhering to the prescribed regimen. Time of saliva collection could also influence the results, especially considering the time elapsed since the last oral hygiene routine. The saliva production volume in response to both tested products should have been analyzed to assess its effect on bacterial concentration. Also the fact that the study was done during a pandemic period with constantly changing restrictions may be a limitation. It is crucial to recognize that the exclusion of 8 participants and the challenges with specimen viability carry implications that affect the statistical strength and the reliability of the results. The final number of participants in this study leads to concerns about the statistical power and the reliability of the results. This reduction was primarily due to participant non-compliance with saliva collection and illness-related exclusions, including COVID-19 and the flu. With fewer participants, the ability to detect statistically significant differences between the experimental and control groups was compromised. A larger sample size would have provided more robust data and a higher confidence in the study’s findings, ensuring that the results could be more generalizable to a larger population​. Another concern is related to the potential differential effects of the tested products on total bacteria concentration, not just *S. mutans*. If one product stimulates more salivation than the other, the bacteria's concentration could be diluted, leading to lower apparent levels. This must be addressed in future studies to ensure that the results accurately reflect the antimicrobial effects of the products rather than variations in saliva production​. A strength of this study was the calibration of investigators during the pre-experiment saliva collection. The post-experiment survey was a useful tool to assess participants' compliance as it would directly influence the outcome. The laboratory qPCR analysis is a reliable method for evaluating the abundance of target cells.

This study highlights the preliminary nature of the findings regarding the antimicrobial effects of CBD-infused lozenges on *Streptococcus mutans*, the small sample size and short duration limit the generalizability of the results. While this study demonstrated the antimicrobial effects of CBD-infused lozenges on *Streptococcus mutans*, it is crucial to consider CBD's broader impact on the overall oral microbiota. The antimicrobial properties of CBD are not limited to *S. mutans*, and there is a possibility that the lozenges could disrupt the balance of beneficial bacteria in the oral cavity. This could affect the oral ecosystem's overall health, potentially leading to unintended consequences. Future studies should investigate how CBD affects the entire oral microbiome, not just targeted pathogens, to ensure the treatment's safety and efficacy in maintaining a healthy oral environment.

The outcomes of this distinctive study are in their initial stages, highlighting the need for more thorough investigations to yield definitive understandings regarding the efficacy of CBD in relation to oral health. These findings introduce the effects of CBD on the oral cavity; however, analysis of more samples is necessary for further exploration and inference. To advance our understanding, future research could consider incorporating microbial analysis as a means to investigate the impact of cannabinoids.
